# Photocatalytic chlorine atom production on mineral dust–sea spray aerosols over the North Atlantic

**DOI:** 10.1073/pnas.2303974120

**Published:** 2023-07-24

**Authors:** Maarten M. J. W. van Herpen, Qinyi Li, Alfonso Saiz-Lopez, Jesper B. Liisberg, Thomas Röckmann, Carlos A. Cuevas, Rafael P. Fernandez, John E. Mak, Natalie M. Mahowald, Peter Hess, Daphne Meidan, Jan-Berend W. Stuut, Matthew S. Johnson

**Affiliations:** ^a^Acacia Impact Innovation, Maarten van Herpen, Bernheze 5384 BB, The Netherlands; ^b^Department of Atmospheric Chemistry and Climate, Institute of Physical Chemistry Blas Cabrera, Spanish National Research Council, 28006 Madrid, Spain; ^c^Department of Chemistry, University of Copenhagen, DK-2100 Copenhagen Ø, Denmark; ^d^Institute for Marine and Atmospheric Research Utrecht, Department of Physics, Faculty of Science, Utrecht University, 3584 CS Utrecht, The Netherlands; ^e^Institute for Interdisciplinary Science, National Research Council, Mendoza 5501, Argentina; ^f^School of Natural Sciences, National University of Cuyo, Mendoza 5501, Argentina; ^g^School of Marine and Atmospheric Sciences, Stony Brook University, Brookhaven, NY 11790; ^h^Department of Earth and Atmospheric Sciences, Cornell University, Ithaca, NY 14853; ^i^Department of Biological and Environmental Engineering, Cornell University, Ithaca, NY 14853; ^j^Royal Netherlands Institute for Sea Research, Landsdiep 4, 1797 SZ, ‘t Horntje, The Netherlands; ^k^Department of Earth Sciences, Faculty of Science, Vrije Universiteit Amsterdam, 1105, Amsterdam, The Netherlands

**Keywords:** methane removal, tropospheric chlorine, chemistry–climate, aerosol chemistry, isotope modeling

## Abstract

Active chlorine in the atmosphere is poorly constrained and so is its role in the oxidation of the potent greenhouse gas methane, causing uncertainty in global methane budgets. We propose a photocatalytic mechanism for chlorine atom production that occurs when Sahara dust mixes with sea spray aerosol. The mechanism is validated by implementation in a global atmospheric model and thereby explaining the episodic, seasonal, and location-dependent ^13^C depletion in CO in air samples from Barbados [J.E. Mak, G. Kra, T. Sandomenico, P. Bergamaschi, *J. Geophys. Res. Atmos.* **108** (2003)], which remained unexplained for decades. The production of Cl can also explain the anomaly in the CO:ethane ratio found at Cape Verde [K. A. Read et al., *J. Geophys. Res. Atmos.* **114** (2009)], in addition to explaining the observation of elevated HOCl [M. J. Lawler et al., *Atmos. Chem. Phys.* **11**, 7617–7628 (2011)]. Our model finds that 3.8 Tg(Cl) y^−1^ is produced over the North Atlantic, making it the dominant source of chlorine in the region; globally, chlorine production increases by 41%. The shift in the methane sink budget due to the increased role of Cl means that isotope-constrained top–down models fail to allocate 12 Tg y^−1^ (2% of total methane emissions) to ^13^C-depleted biological sources such as agriculture and wetlands. Since 2014, an increase in North African dust emissions has increased the ^13^C isotope of atmospheric CH_4_, thereby partially masking a much greater decline in this isotope, which has implications for the interpretation of the drivers behind the recent increase of methane in the atmosphere.

## Significance

Using a combination of field data and global modeling, we demonstrate a mechanism in which a mix of Sahara dust and sea spray aerosol activated by sunlight produces large amounts of active chlorine. This mechanism resolves a number of unexplained observations and significantly revises our understanding of atmospheric chlorine, reducing uncertainties in the source budget. The chlorine formed by this mechanism impacts two important greenhouse gasses, methane and tropospheric ozone, with an estimated catalytic efficiency of removing ca. 45 methane molecules per iron atom per day. The inclusion of Cl^.^ made from the photocatalytic oxidation of ocean chloride in models will reduce critical uncertainties in estimates of methane emissions and improve our ability to predict future climate change.

Cycling of chlorine from the oceans through the atmosphere impacts climate and air quality (1). These processes are driven by highly reactive atomic chlorine, Cl. Chlorine atoms can initiate the breakdown of methane, a well-mixed greenhouse gas responsible for >1/3 of global warming since preindustrial times (2) whose atmospheric burden continues to increase with a new concentration record set in 2022 (3). While chloride ions (Cl^−^), the reduced form, are common in sea spray aerosol, only a few processes can oxidize them to atomic Cl, which has such low concentrations (10^3^ to 10^4^ cm^−3^) that it cannot be detected directly.

A sensitive and selective indirect quantification of the concentration of atomic Cl makes use of the strong carbon kinetic isotope effect (KIE) in the CH_4_ + Cl reaction (4–7). The KIE of OH oxidation of CH_4_ is around 3.9‰ at room temperature (4), while the KIE for Cl oxidation is 66‰ (5). ^12^CH_4_ reacts more quickly than ^13^CH_4_, leaving the remaining CH_4_ enriched in ^13^C (4–7) and producing extremely ^13^C-depleted CO (8). If a larger fraction of CH_4_ is oxidized by Cl, this will lead to a larger enrichment of ^13^C in CH_4_. Globally, there are substantial differences between models’ estimates of the fraction of methane removed by Cl, ranging from 0.8 to 3.3% (9–13). A study by Wang et al. (14) found additional oxidized chlorine in field studies relative to the levels predicted by models and conclude that, if correct, there is a very large missing primary source of Cl atoms.

The observed abundance of ^13^C in tropospheric methane is used to constrain the sources of methane using their characteristic δ^13^C-CH_4_ values. Uncertainties in gas-phase Cl concentrations (“[Cl]”) propagate into methane source budgets through isotope-constrained top–down models (15–20). For CH_4_, the ^13^C/^12^C ratio is reported using the delta notation δ^13^C defined by δ^13^C = ([^13^CH_4_]/[^12^CH_4_] – *R_0_*)/*R_0_*, where *R_0_* = (^13^C/^12^C)_PDB_ has a value of 0.0112372 for the isotope standard, Pee Dee belemnite (PDB) (6). Delta values are normally multiplied by 1000 and presented in units of per mil *‰*. Biological methane sources such as wetlands, ruminants, and rice production emit CH_4_ with a δ^13^C-CH_4_ value in the range of −65 to −55‰, depleted in ^13^C relative to fossil fuel and biomass burning which range from −45 to −25‰. These emissions mix leading to an emission average δ^13^C-CH_4_ of, for example, −54.3‰. However, this is not the value seen in the atmosphere because its composition is also changed by the OH and Cl sinks. Due to the KIE, oxidation of CH_4_ by OH and Cl enriches the remaining CH_4_ in ^13^C, leading to an observed δ^13^C-CH_4_ value of, for example, −47.1‰, thus making the observed δ^13^C-CH_4_ seem to include less biological methane sources than what was actually emitted. Here, we present a significant chlorine source, and if it is not included in isotope-based emission budgets, these models will underestimate the biological methane source. The Cl-KIE makes top–down models especially sensitive to the Cl + CH_4_ reaction; each 1% increase in CH_4_ loss via Cl increases mean δ^13^C-CH_4_ by ~0.5‰ (18). To put this in perspective, the recent rapid increase of atmospheric methane (3) is associated with a change in the observed δ^13^C-CH_4_ from −47.1‰ in 2007 to −47.5‰ in 2022 (21). A better understanding of the Cl sink of CH_4_ will significantly improve our understanding of the sources responsible for the recent CH_4_ increase. If current models underestimate the source of Cl, they would compensate by underestimating the relative contribution from ^13^C-depleted biological sources and overestimating the contribution of ^13^C-rich fossil sources (19, 20).

Anomalous results from a number of field studies could be explained using a so-far-unknown, episodic source of Cl. Read et al. measured the intra-annual cycles of nonmethane volatile organic compounds and other species at Cape Verde and found that CO and ethane, which have similar lifetimes with respect to OH degradation, vary intra-annually with the sinusoidal variability expected due to their reaction with OH but with CO showing a smaller cycle amplitude (22). This is unusual because the OH sink will not discriminate between CO and ethane, so the ratio of CO to ethane should be stable. While Read et al. suggest that the anomaly in the CO:ethane ratio is due to substantial chlorine chemistry, there was no known chlorine source that would operate along the entire 50-d lifetime from source to detection, and which has significant seasonal variation. In separate work, Lawler et al. measured HOCl in marine air at Cape Verde and found unusual HOCl levels that could be explained only by adding an unknown Cl source that is at 100 to 1,000 times stronger than conventional acid displacement (23). Finally, Saharan dust has been observed to affect O_3_ in the free troposphere at the Monte Cimone Climate Observatory, with implications for surface air quality in cities of northern Italy and southern Spain (24).

Here, we present field and modeling evidence of the mechanism of the production of atomic Cl via the photocatalytic oxidation of chloride in aerosols containing Sahara mineral dust. By this mechanism, Cl_2_ and Cl are generated when lofted iron-bearing mineral dust aerosol from North Africa descends into the marine boundary layer (MBL) over the Atlantic and mixes with sea spray aerosol to form Mineral Dust-Sea spray Aerosols (MDSA). We combine data from field with global atmospheric modeling and predict extremely low δ^13^C-CO values that match those seen in CO in air samples from Barbados (25); these results remained unexplained for 20 y. Finally, we discuss the global significance of this mechanism that is not yet included in global models.

## Results

### Barbados Observations.

**Fig. 1.**
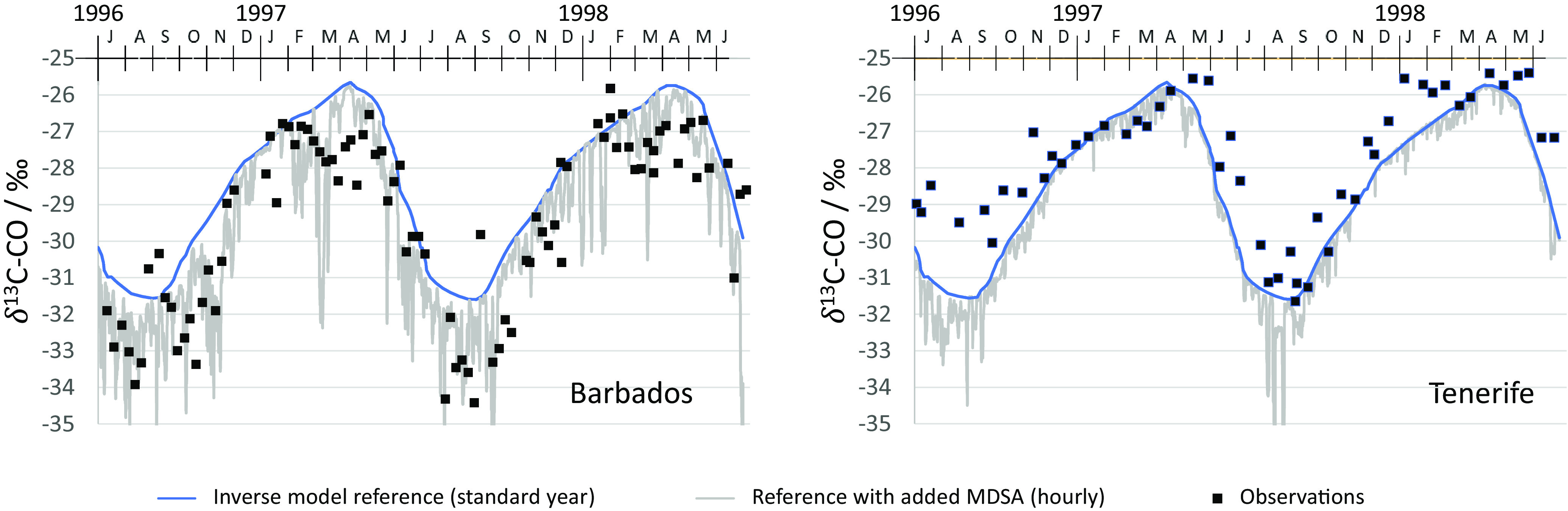
Time series showing δ^13^C-CO according to the CESM simulations including the MDSA mechanism. Barbados (*Left*) and Tenerife (*Right*; hourly; gray line), together with an inverse box model simulation based on traditional chemistry for a standard year as used by Mak et al. (25) (blue line, see *Methods*). The model reproduces the exceptionally negative excursions in the δ^13^C-CO data from Mak et al. (25) (boxes).

Mak et al. (25) saw the largest ^13^C depletions in CO ever recorded, in air samples taken on Barbados (13.2 °N, 59.5 °W) from 1996 to 1999 (Fig. 1). The Barbados samples were depleted in ^13^C by up to −3‰ relative to the other northern hemisphere locations in episodes occurring from July to December, reducing the seasonal minimum δ^13^C-CO at Barbados to −34‰. For other locations, for example, Tenerife (28.3 °N, 16.5 °W), the seasonal minimum of δ^13^C-CO remained above −31‰ (Fig. 1). As seen in the fig., the seasonal maximum at Barbados was similar to that seen at the other locations, around −26‰. Thus, the depletion events are seasonal, episodic, and location specific.

Mak et al. (25) suggest the observed changes in ^13^CO and C^18^O are evidence of the reaction of Cl with CH_4_. CO is the first stable product in atmospheric methane oxidation, and δ^13^C-CO is sensitive to even small CH_4_ removal by Cl because atmospheric (CO) is more than an order of magnitude lower than [CH_4_] (8) and because of the large KIE of the Cl reaction. They estimated that the data could be explained if 3 ppbv of CH_4_ were destroyed by reaction with [Cl] = 5 × 10^4^ atoms cm^−3^ over 3 d. However, the known Cl sources could not explain the seasonal depletion in ^13^CO (25).

We incorporated the MDSA mechanism into the global 3-D chemistry-climate model Community Atmosphere Model with Chemistry (CAM-Chem) (*Methods*) (v4) to investigate whether Cl generated by MDSA photocatalysis could be responsible for the CO isotope signal observed by Mak et al. (25). CAM-Chem is included in the well-documented CESM (Community Earth System Model) framework (26, 27) and includes an updated scheme for halogen chemistry (Cl, Br, and I) (13). The parameters used in the model simulation are based on laboratory and field observations from the North Atlantic region near Barbados, which makes it useful for describing the CO isotope anomaly at Barbados, although interpretation in other regions may be less reliable.

Output from our MDSA model shows good agreement with the timing, episodic variability, and magnitude of δ^13^C-CO observations of Mak (Fig. 1). We will discuss this in detail after we first introduce the mechanism.

### The Mechanism of Chlorine Production by Sahara Dust Photochemistry.

MDSA is formed when iron-bearing mineral dust and sea spray aerosols mix (28). Iron is released from the minerals by weathering, and according to the Pourbaix diagram (29), iron oxides and hydroxides, abundant in the crust and in mineral dust, are converted into Fe(III) ions under the oxidizing and acidic conditions found in well-mixed marine and dust aerosols found in the North Atlantic (30). Acidity favors formation of Fe(III) chlorides (FeCl*_n_*^3-^*^n^* with *n* = 1..4) by keeping hydroxide concentrations low (31). Fe(III) chlorides have an intense ligand to metal electron transfer absorption activated by sunlight, yielding Fe(II) and oxidized chlorine Cl (14). The Cl atoms can escape directly from the aerosol to the gas phase but most likely combine to escape as Cl_2_, which is then quickly photolyzed by sunlight (31). Fe(II) is reoxidized to Fe(III) by O_3_, H_2_O_2_, and other species, making the process catalytic in iron by reforming the iron chloride species (29). When Cl reacts with methane, it forms HCl, which can be reabsorbed by the MDSA, making the process catalytic in Cl as well (see *SI Appendix*, Fig. S1 for a scheme of the mechanism). While this mechanism resembles the release of iodine from dust reported by Koening et al. (32), in this case, the halogen source is the large reservoir of chloride from sea spray already present in the aerosol phase in contrast to deposition of iodine-containing atmospheric gases on the mineral surface. This critical difference means that MDSA–chlorine is produced close to the surface in the MBL from abundant sea spray aerosol. Using a smog chamber, Wittmer et al. (31) demonstrated that formation of Cl atoms by photooxidation of chloride occurs in aerosols that form when iron oxide is mixed with sea salt under acidic conditions. Until now, this mechanism has not been observed in situ and its global impact was unknown, although the necessary elements are found in the troposphere: Photoactive iron is known to exist above the North Atlantic (33–35) and mineral particles in the MBL accumulate oxidation products and salts (including sea salt) on their surface within a day (28).

Zhu et al. (34) collected mineral aerosol at Barbados and measured the daily variability of Fe(II). They saw a clear diurnal pattern in the concentration of soluble Fe(II), with daytime values (mean 3.7 ng m^−3^) about twice nighttime values (1.5 ng m^−3^), showing that photochemical processing of Fe takes place. The same researchers found that a photochemical steady state is established in which Fe(III) is photoreduced to Fe(II) as quickly as Fe(II) is oxidized to Fe(III) (33), and they showed that there are at most 11.4 Fe(II)-Fe(III) cycles h^−1^ at maximum solar intensity. This rate is at the low end of the range reported by Wittmer of 6 to 78 h^−1^ (31); atmospheric conditions could result in lower values, more similar to those assumed here, due to the suppression of this reaction in the presence of environmental sulfate (36). Several mechanisms for the photochemical reduction of Fe(III) are known including the oxidation of organics yielding H_2_O_2_ (33), but we assume in our model parameters that the dominant photoreduction reaction in MDSA yields OH and/or Cl that both lead to Cl_2_ production (31). Considering that these dust particles will be mixed with sea salt and assuming the irradiated combination gives rise to oxidized chlorine via the MDSA mechanism, each cycle would produce one Cl atom. The agenda is to i) determine whether Fe photochemistry in Sahara dust is able to produce Cl atoms, ii) model the MDSA mechanism over the North Atlantic, iii) compare the model prediction with the observed CO isotope anomaly in Barbados, and, if successful, iv) estimate the global impact of MDSA photochemistry.

### Model Results.

The MDSA mechanism is implemented in the CESM assuming: i) The cycling rate observed near Barbados between Fe(II) and Fe(III) is fully equated with the rate of Cl production, and ii) the influence of sulfate is reflected in these observations because FeSO_4_ (which would reduce the Fe available for Cl production) does not take part in the observed photochemistry cycles (36). We ran the model with and without MDSA chemistry and used a tracer molecule to derive the additional CO generated by the Cl + CH_4_ oxidation due to the addition of MDSA. We use this to calculate the change in δ^13^C-CO and add the result to the same inverse model as was used by Mak et al. (25) (see *Methods* for details). We then evaluate how this affects the predicted seasonal minimum and maximum of the inverse model.

The model results show that MDSA photocatalysis produces 110 ± 10 Cl atoms (active Fe atom)^−1^ d^−1^ on average at Barbados, which is 70 g Cl_2_ (g active Fe)^−1^ d^−1^. The modeled average cycling rate between Fe(II) and Fe(III) of 11 h^−1^ at noon, is similar to that of the observations, consistent with the implemented reaction rates observed by Zhu (33). According to the model results, the average concentration of photoactive iron in MDSA at Barbados is 2.5 ng m^−3^ with peak concentrations up to 34 ng m^−3^ [similar to observations reported by Zhu (34)], resulting in a Cl_2_ production rate of up to 6 × 10^5^ cm^−3^ s^−1^ (average 2 × 10^4^ cm^−3^ s^−1^) and peak [Cl] of >10^5^ cm^−3^.

The model output shows good agreement with observation (25) for the location, timing, episodic variability, and magnitude of δ^13^C-CO (Fig. 1). The MDSA model reduced the seasonal minimum of weekly average δ^13^C-CO at Barbados by −2‰ in 1996 and by −4‰ in 1997. Because of the episodic nature of the dust intrusions, the springtime dust does not significantly change the seasonal maximum, changing it by only −0.1‰ and −0.3‰ in 1996 and 1997, respectively. The large episodic variations in δ^13^C-CO at Barbados are also seen in the observations. In our model, at Spitzbergen, both the seasonal minimum and maximum did not change by more than −0.3‰, confirming that the MDSA impact is local and specific to the western North Atlantic. Cl formation in the model depends on specific conditions, including dust concentration, wind speed/sea spray aerosol loading, atmospheric circulation, wind direction, and actinic flux. Mak et al. (25) suggested that chlorine is the cause of the ^13^C anomaly in CO, and our CESM simulation is consistent with our hypothesis that the Cl is associated with Sahara dust episodes.

Our model results show that seasonal Cl_2_ production rates up to 6 × 10^5^ cm^−3^ s^−1^ are needed to explain the CO isotope anomaly at Barbados. Other known Cl production mechanisms are not able to produce a seasonality of this magnitude. For example, acid displacement in sea salt forms gaseous HCl which can then react with OH becoming a large source of Cl above the oceans, Cl (14, 37). We estimate that the maximum Cl production from this source is 3 × 10^4^ cm^−3^ s^−1^ (*Methods*), which is more than an order of magnitude too small to explain the observations. In addition, the global seasonality of acid displacement as a Cl source comes from solar-intensity-driven OH and DMS seasonality (6), which makes acid displacement seasonality more apparent away from the equator, and therefore does not match with the uniqueness of the isotope anomaly at Barbados. Finally, nonsea salt sulfate levels (and associated HCl levels) at Barbados reach their seasonal maximum in June/July (38), which is opposite to the observed CO isotope effect. Other substantial Cl sources such as ClO and HOCl have at most a similar order of magnitude and are part of the Cl radical chain with their seasonality depending on an initial input of Cl from sources like OH + HCl. We therefore conclude that MDSA photocatalysis is the necessary mechanism for producing Cl and with the right concentration and spatial and temporal structure to explain the observations.

As was already observed by Mak et al. (25), the observations in Barbados are depleted compared to the observations in Tenerife (Fig. 1). In agreement with this observation, the model predicts much smaller MDSA-related dips in δ^13^C-CO at Tenerife (Fig. 1*B*) and less frequently. Braunlich (39) discussed the Tenerife ^13^CO record in detail and noted that the summer minimum in Tenerife was lower in 1997 compared to 1996, 1998, and 1999. This was also observed in stations at Spitzbergen and Sonnblick, but no explanation could be found. This period aligns with the prediction by our model of significant Cl production by the MDSA mechanism in Tenerife for August 1997.

**Fig. 2.**
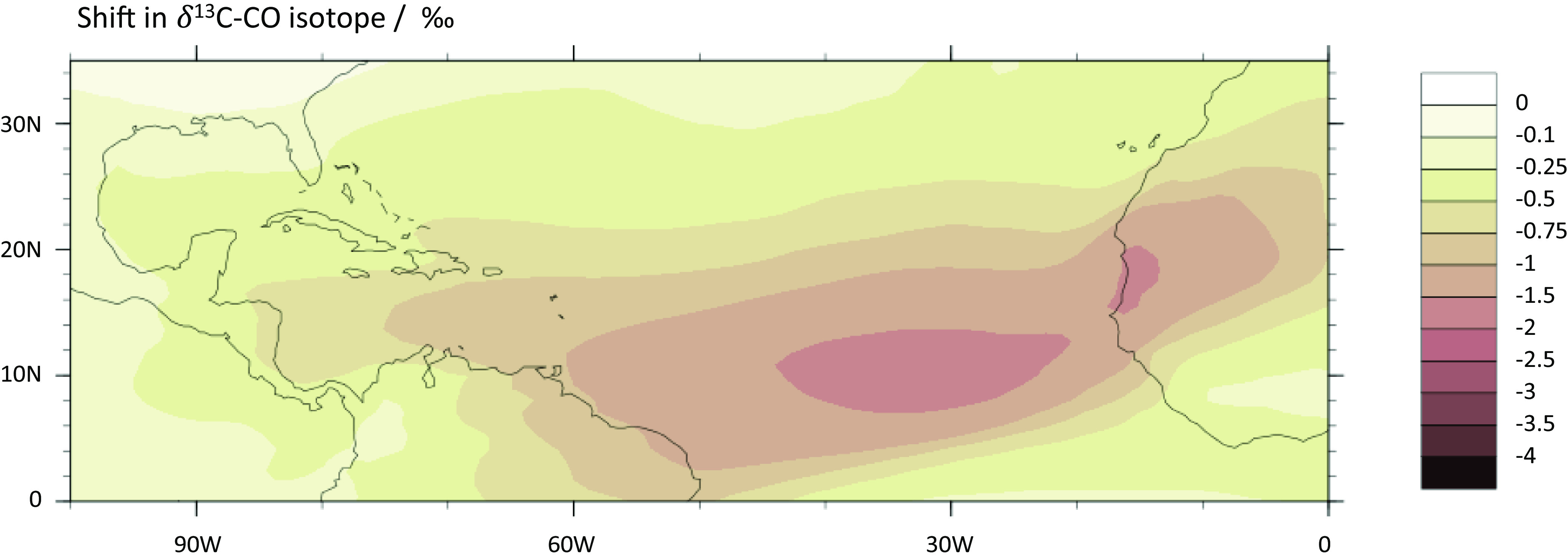
Map of the North Atlantic region showing the annual average shift in δ^13^C-CO in the MBL due to Cl generated by MDSA in the CESM model simulations. By running the model with and without the MDSA mechanism, we calculated the change in δ^13^C-CO by the additional CO produced from the Cl-CH_4_ reaction.

The modeled annual average shift in δ^13^C-CO across the North Atlantic region shows a shift of up to −4‰ at 30 °W over the North Atlantic (Fig. 2; monthly in *SI Appendix*, Fig. S2). The zone of maximum shift changes through the year depending on winds in the Inter-Tropical Convergence Zone that carry Sahara dust to Barbados from June through December. Even though more dust is transported across the Atlantic in summer, the model predicted that the strongest δ^13^C-CO excursions at Barbados would occur in the fall as is observed. This is because in summer, most dust is transported at high altitude, above the MBL, which limits mixing with sea spray aerosol (*SI Appendix*, Fig. S3). This agrees with satellite observations (40) and in situ measurements (41) that show that the Saharan Air Layer transports dust at a higher altitude and velocity in summer than fall. Depletion in ^13^C-CO is also seen in the spring (Fig. 1). While Sahara dust is farther south of Barbados this time of year, its impact is still strong enough to influence δ^13^C-CO at Barbados. However, because of the episodic nature of the dust intrusions, the springtime dust photocatalysis does not significantly change the seasonal maximum.

**Fig. 3.**
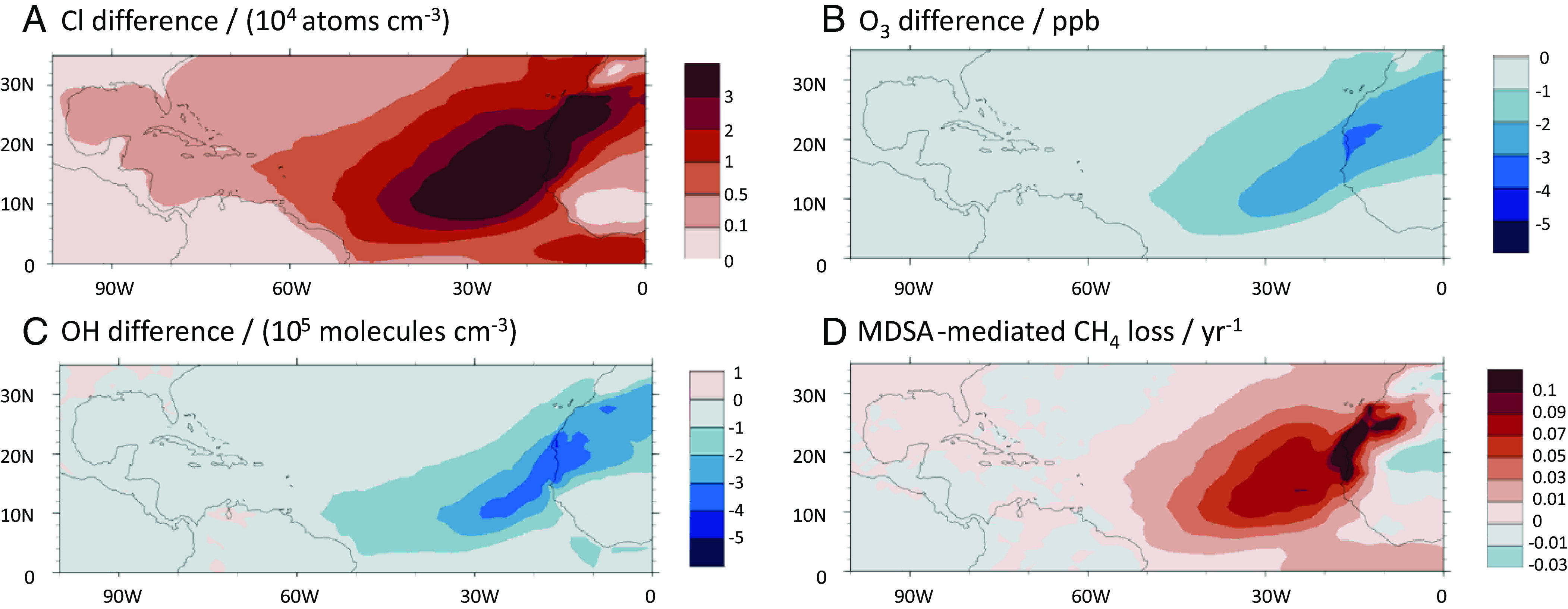
Spatial pattern of the annual average changes in the MBL in (*A*) [Cl], (*B*) Δ*x*_O3_, (*C*) [OH], and (*D*) CH_4_ loss rate due to modeled MDSA photochemistry, showing how MDSA photochemistry impacts CH_4_ through its effects on OH and Cl. *SI Appendix*, Figs. S5–S8 show model outputs without MDSA, with MDSA, and relative difference for each of these four parameters.

MDSA photochemistry increases the monthly average [Cl] to >10^4^ cm^−3^ over the equatorial Eastern Atlantic (Fig. 3*A*) greatly increasing the fraction of CH_4_ oxidized by Cl. Fig. 3*B* shows the reduction in O_3_, which is a precursor for OH and leads to a reduction in OH shown in Fig. 3*C*. The overall effect on CH_4_ loss is the sum of the additional loss via Cl and the reduced loss via OH. For most of the North Atlantic, we find that CH_4_ removal increases with the addition of the MDSA mechanism (Fig. 3*D* and *SI Appendix*, Fig. S7); the local CH_4_ loss rate increases by 20%, even though OH is reduced by up to 10% (Fig. 3*C* and *SI Appendix*, Fig. S6).

For the North Atlantic region (0 °N to 35° N; 90° W to 0° W), an annual average burden of 0.22 Gg photochemically active Fe in MDSA in the MBL above the ocean surface resulted in a Cl_2_ production of 2.9 Tg y^−1^ [36 g Cl_2_ (g active iron)^−1^ d^−1^], resulting in a net CH_4_ loss of 0.6 Tg y^−1^ [7.5 g CH_4_ (g active iron)^−1^ d^−1^], from an increased Cl-mediated CH_4_ loss of 1.1 Tg y^−1^ and a reduced OH-mediated loss of 0.5 Tg y^−1^ (*SI Appendix*, Table S1). The total burden of tropospheric O_3_ was reduced by 6% above the North Atlantic (*SI Appendix*, Table S1).

These model results suggest that MDSA produce a large amount of Cl_2_ that is not considered in existing Cl budgets (11): 3.8 Tg Cl_2_ y^−1^ across the entire North Atlantic (continental and marine), of which 2.9 Tg y^−1^ are over marine areas. Globally, the MDSA Cl source increased the tropospheric inorganic Cl source in our CESM simulations by ~41%, adding 13 Tg y^−1^ of chlorine to 23 Tg y^−1^ from sea-salt cycling, 8 Tg y^−1^ from anthropogenic emission, and 1 Tg y^−1^ from photochemical oxidation of organic chlorine. Because the MDSA source is very strong over the North Atlantic, this increases the Cl contribution to CH_4_ oxidation by at least fourfold, making the MDSA mechanism the dominant source of atomic chlorine in this region. Outside of the North Atlantic region, our model predicted Cl_2_ production by MDSA in dust from the east coast of Argentina, as well as in the Gulf of Aden, around India and around Australia.

## Discussion

Li et al. (13) found that the effect of chlorine in the atmosphere today is to reduce OH production and increase its loss particularly in the free troposphere and over the open ocean, leading to a net increase in the CH_4_ burden. In this work, using the same model, we obtain the same initial result for the global average behavior. In addition, we see that atmospheric behavior changes during intense MDSA episodes: Chlorine production continues to remove methane after tropospheric ozone levels (the precursor for OH) have been reduced. Thus, after an initial increase, there is a significant decrease in methane lifetime in regions with high Cl concentrations [see Fig. 3*B* for the regional depletion of O_3_, and Fig. 3*D* for the regional decrease in methane lifetime, and also *SI Appendix*, Fig. S10). A second reason for significant methane oxidation by Cl in the North Atlantic is that the CESM model shows elevated NOx in this region (yearly average surface level mixing ratio of 221.1 pptv (without MDSA case) and 216.1 pptv (with MDSA case)] relative to the global average (146.7 pptv for no-MDSA and 144.7 pptv with MDSA). This is due to an increased NO_x_ source in the northern hemisphere from human activity and long-range transport via PAN-type compounds. This NO_x_ helps prevent OH loss and increases the radical chain length (1). The extra O_3_ loss in our model simulation with MDSA was driven by chlorine-initiated reactions [determined by categorization of the O_3_ loss channels in the North Atlantic MBL following (42)]. The significant reduction in O_3_ in the MBL resulted in a reduction in bromine and iodine emission and their atmospheric abundance, leading to a slight reduction in the O_3_ loss channel for Br*y* and I*y.* The effects of the specific families can be seen in *SI Appendix*, Table S3.

Many atmospheric species are sensitive to changes in chlorine. Future research may be able to show that the MDSA mechanism can explain a variety of phenomena in particular at the regional scale, including ozone suppression, VOC oxidation, VOC oxidation-driven secondary organic aerosol formation, HCl formation, etc. Additional Cl from MDSA would be able to explain the variation in the CO:ethane ratio observed by Read et al. (22), and an additional chlorine source is consistent with Lawler et al.’s observation of elevated Cl_2_ and HOCl in air masses arriving at Cape Verde (23). Lawler observed HOCl up to 100 ppt at Cape Verde and estimated that the unknown Cl source responsible for the increase in HOCl is at least one order of magnitude greater than acid displacement of HCl, which is in agreement with the magnitude that we found in this study. Over the North Atlantic, the monthly average HOCl levels in our model output were increased from 0.3 to 2.0 pptv (without MDSA) to 0.9 to 52.1 pptv (with MDSA), during our simulation period. Cl from MDSA reduces the annual average tropospheric ozone burden in the North Atlantic MBL by 6%. The substantial local MDSA-mediated ozone loss helps to reconcile observations and models by providing a mechanism for dust-induced tropospheric ozone depletion in air masses rich in mineral dust from the North African desert (43). Our model includes the stratosphere but found no impact on stratospheric ozone.

Our model showed that Cl from mineral dust/salt aerosols increases CH_4_ loss via Cl by 4.8 Tg y^−1^ globally (0.9% of total CH_4_ loss). This is important given the aforementioned uncertainty in estimates of the fraction of CH_4_ removed by Cl (9–14, 37). The additional 0.9% Cl oxidation of CH_4_ shifts global δ^13^C-CH_4_ by ~0.5‰ (18). Adding this into top–down methane emission models shifts 12 Tg y^−1^ of estimated CH_4_ emissions (2% of the global total emissions) toward ^13^C-depleted biological sources such as agriculture, potentially reducing the estimated fraction of fossil-fuel methane emissions by 7% and increasing the ruminant contribution by a corresponding amount (19).

**Fig. 4.**
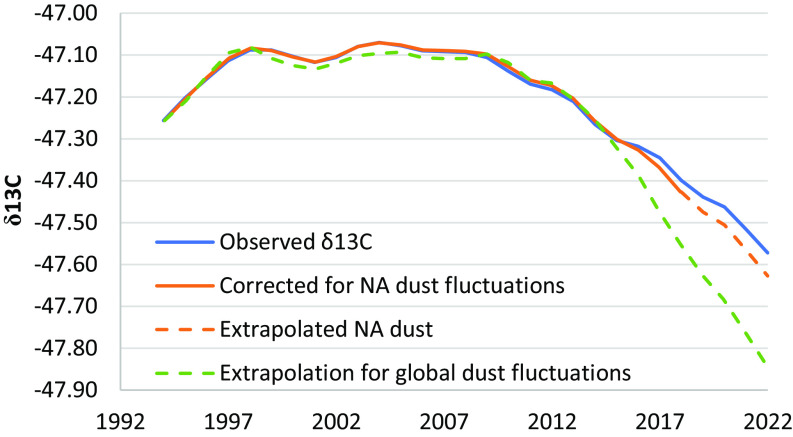
The impact of dust fluctuations on the observed δ^13^C-CH_4_. The figure shows what the observed δ^13^C would have been if North African dust had remained stable. Recent increases in dust from North Africa resulted in additional Cl oxidation of methane. This increased δ^13^C, and in this way masked a much larger change in the isotope. The green dashed line shows an extrapolation that assumes that global MDSA will follow the trend in North African dust (43, 44).

A doubling of this large change in methane isotopes from the MDSA mechanism is likely to have occurred over the 20th century since available in situ and paleoclimate data suggest an almost-doubling of the transport of North African dust into the North Atlantic, especially since 2014 (43, 44). The large changes in atmospheric dust loading seen over geological time scales show that it is highly variable. Another study predicts a 60% reduction in dust from the Holocene to today (45). These changes could be expected to affect chlorine production via the MDSA mechanism. Depending on the conditions where the chlorine is added, this could modestly increase or decrease methane lifetime. Perhaps more importantly, enhancing the Cl sink of methane shifts our estimates of methane sources. It is thus extremely important to accurately quantify this source as part of efforts to understand and control atmospheric methane. Using a box model, we calculated the change in the atmospheric ^13^CH_4_ isotope (21) in response to recent changes in North African dust emissions, by scaling the Cl production of MDSA linearly with the reported dust emission (44). Fig. 4 shows that these fluctuations in dust emission give ^13^CH_4_ isotope changes that are significant compared to typical yearly changes (16, 17) and could in that way mask changes to emission sources. In conclusion, the MDSA chlorine source should be verified in future laboratory and field studies and should be included in methane emission models to improve their accuracy, and further studies should be done to evaluate the importance of the MDSA mechanism in other regions.

## Materials and Methods

### CESM Model Setup.

The model employed in this work is a global chemistry–climate model CAM-Chem, which is included in the well-documented CESM framework (CESM) (26, 27), and incorporates an updated halogen chemistry scheme for chlorine, bromine, and iodine (14). In this work, CAM-Chem was configured with a horizontal resolution of 0.9° latitude by 1.25° longitude and 56 levels from the surface to the stratosphere.

### MDSA Chemistry Implementation in CESM.

For this implementation of the MDSA, we use a simplified mechanism to describe the overall process based on results from field studies. At present, there is no model that combines iron aerosols and chlorine chemistry to describe the elementary processes in detail. In our parameterization, the amount of MDSA was calculated by estimating the amount of photoactive iron. In studies of Fe(II) and photoreducible Fe(III) in North Atlantic dust aerosols, it was observed that photoreducible Fe(III) is found mainly in fine dust particles of sizes below 2.5 µm and across a wide range of dust loads (46–48). These observations imply that a substantial fraction of these dust/salt aerosols are in the acceptable pH range of the Pourbaix diagram (29) for the reoxidation of Fe(II) to Fe(III). We used these observations as input for our model. We used the size bin of 1 to 2.5 µm from the model (referred to as χ_DST02_ below) because at least 90% of Sahara dust <2.5 µm is in the 1 to 2.5 µm range, and χ_DST02_ therefore provides us with a good reference value to compare with the observations. Additionally, the smallest size fraction is often associated with high sulfate concentrations (30), which could suppress the MDSA (49). In situ observations suggest that most fine particles downwind of the Saharan plume will be acidic enough for the MDSA mechanism (30, 50).

The following equation was used to calculate the amount Fe in MDSA.

[1] $$w_{\mathrm{MDSA}}=\alpha \cdot c\left(\chi_{DST02}\cdot \gamma \right),$$

where α = 0.063% is the mass fraction of photoactive Fe in the dust, *c* is a pressure-dependent factor converting χ from kg kg^−1^ in the CESM model to kg m^−3^, and γ is a parameter set to 1 or 0 depending on the availability of Cl as described below.

This mechanism should be active only in downwind regions from dust plumes and in the MBL since this will assure a high-enough Cl:Fe ratio, above 13 for MDSA concentrations up to 90 ng m^−3^ (31). In order to ensure these characteristics, we limit the Cl production using the constraints ρSSLT02 > 10^−9^ and altitude below 900 hPa, by setting γ = 1, and otherwise setting γ = 0. Mineral particles in the MBL accumulate oxidation products and salts including sea salt on their surface (28), with 80 to 90% of the dust particles becoming mixed with sea salt within a day.

The factor α is calculated by multiplying the fraction of Fe in the dust (3.5%) with the fraction of the iron that is soluble and photoactive (1.8%). We used 3.5% as the amount of iron in the Saharan dust, based on the findings of Trapp et al. 2010 (48), as a typical value for dust-sea salt mixtures across the North Atlantic.

Observational evidence suggests an inverse relationship between dust amount and solubility (51). The two explanations for this behavior are 1) differences in particle size (smaller combustion-sourced particles having higher solubility than dust-sourced particles) and 2) downwind atmospheric processing of particles increasing the solubility and the higher gravitational settling rate of larger particles (52). However, the fraction of the total iron that is photoactive does not appear to follow this inverse relationship at locations downstream from the dust sources (48) because observations across the North Atlantic show a similar fraction of total Fe to be photoactive, for both high and low dust loads (46–48). Therefore, we assume a constant value for the fraction of the iron that is photoactive for the long range–transported dust of 1.8% based on cruises far from North Africa (35, 46–48) and average values for the model-data syntheses for dust in the North Atlantic (52). Our parameter of 1.8% photoactive soluble iron is in line with the findings reported by Zhu et al. (34) who measured the daily variability of Fe(II) at Barbados in September 1992 and reported a clear diurnal pattern in the concentration of soluble Fe(II), with daytime values (mean 3.7 ng m^−3^) about twice nighttime values (1.5 ng m^−3^). This observed cycling of Fe(II) and Fe(III) during the day and night demonstrated that photochemical processing of Fe does take place at Barbados, and we postulate here that it is entirely due to the Cl mechanism. Next, the MDSA number *w_MDSA_* is converted to *d_MDSA_* in mol Fe m^−3^, using M_w_(Fe) = 55.845 g mol^−1^.

[2] $$\begin{array}{c} d_{\mathrm{MDSA}}\left(\mathrm{mol}m^{-3}\right)=w_{\mathrm{MDSA}}\left(\mathrm{kg}m^{-3}\right)\cdot \frac{1,000g{\mathrm{kg}}^{-1}}{55.845g{\mathrm{mol}}^{-1}}. \end{array}$$

Zhu et al. (33) studied Fe(III) photochemistry for typical (aerosol) conditions at Barbados. They found that for constant solar intensity, a steady-state is established in which Fe(III) is photoreduced to Fe(II) as quickly as Fe(II) is oxidized to Fe(III) by H_2_O_2_. They demonstrated experimentally that in a typical aerosol at Barbados, the oxidation of Fe(II) to Fe(III) by H_2_O_2_ has a reaction rate constant of 0.19 min^−1^ at a typical H_2_O_2_ concentration of 50 µM at pH = 1. They also showed that during most of the day, this balance remained stable at high Fe(II) levels, which indicates that photoreduction is much faster than oxidation. Using the reaction rate of 0.19 min^−1^ would allow for a maximum of 11.4 photochemical cycles h^−1^ if photoreduction is not the limiting step. This is in good agreement with the values found by Wittmer (31) of 6 to 78 h^−1^, although the Fe-Cl reaction is sensitive to chemical composition and is partly suppressed in the presence of sulfate (33).

The MDSA aerosol chemistry is described by Wittmer (36, 49). Fe(III) chlorides FeCl*_n_*^3-^*^n^* (*n* = 1..4) have intense UV charge-transfer absorptions. On absorption of light, an electron is transferred from chloride to Fe(III), resulting in photoreduction of Fe(III) to Fe(II) and yielding chlorine atoms (Cl) in the liquid phase. Chlorine atom recombination is catalyzed by Cl^−^ via Cl_2_^−^ leading to Cl_2_ which degasses.

In summary, Cl production occurs at the rate at which iron is cycled between Fe(II) and Fe(III) within *d_MDSA_*. Assuming steady state, the fraction of *d_MDSA_* that is in the Fe(II) state is given by

[3] $$\mathrm{Fe}{\left(\mathrm{II}\right)}_{fraction}=\frac{k_{2}\frac{I}{I_{o}}}{k_{1}+k_{2}\frac{I}{I_{o}}},$$

In which *k_1_* is the reaction rate constant for oxidation of Fe(II) to Fe(III), and *k_2_* is the reaction rate constant for photoreduction of Fe(III) to Fe(II) at solar intensity *I_0_* = 600 W m^−2^, and *I* is the temporally varying solar intensity.

At steady state, the production of Cl can be calculated from either Fe(II) oxidation or Fe(III) photoreduction. Using Fe(II) oxidation, the Cl production rate (amount per unit time; $r_{\mathrm{Cl}}\;$ ) is

[4] $$\begin{array}{c} r_{\mathrm{cl}}=\frac{d[\mathrm{Fe}(\mathrm{II})]}{\mathrm{dt}}=MDSA_{\mathrm{mol}}\cdot k_{1}\cdot \frac{k_{2}\frac{I}{I_{o}}}{k_{1}+k_{2}\frac{I}{I_{o}}}. \end{array}$$

Combining Eqs. **1** to **4** and using two Cl atoms to create one Cl_2_ molecule gives the following rate of Cl_2_ production:

[5] $$\begin{array}{c} r_{c12}=\frac{500}{55.845}\alpha \cdot c\left(\chi_{DST02}\gamma_{02}\right)\cdot \frac{k_{1}k_{2}\frac{I}{I_{o}}}{k_{1}+k_{2}\frac{I}{I_{o}}}\mathrm{mol}m^{-3}\min^{-1}. \end{array}$$

For oxidation, we used the reaction rate constants from Zhu et al. (30), *k_1_* = 0.19 min^−1^. Because field measurements suggest that photoreduction is not the limiting factor, we used a photoreduction reaction rate of 10 times as fast: *k_2_* = 1.9 min^−1^. The resulting Cl_2_ production rate is in min^−1^ as well. These values of *k_1_* and *k_2_* will result in a maximum of 10.2 photochemistry cycles per hour at maximum sunlight, which is well within the range of cycles measured by Wittmer (31) of 6 to 78 Cl h^−1^.

### Analysis.

To monitor the reaction of Cl + CH_4_, we added a tracer to the model with a lifetime of 60 d, representative of the lifetime of CO in the North Atlantic region, i.e., 0° N to 35° N; 90° W to 0° W), based on Pfister et al. (53). We then ran the model with and without MDSA chemistry, using a stabilization time of 12 mo. Using a CO yield of 90%, the difference in the tracer molecule represented the extra CO generated by the Cl + CH_4_ oxidation due to MDSA implementation.

To calculate the isotope shift in the CO produced via CH_4_ + Cl, we used 100‰ depletion in ^13^C relative to the ambient CO without the MDSA mechanism. The MDSA implementation also changed the amount of CO produced by CH_4_ + OH slightly; the isotope shift from this effect was less than 0.05‰ for a 1 ppb change in CO, calculated using the Rayleigh fractionation equation, and was therefore further neglected in the analysis.

### Sampling at Barbados and Inverse Modeling of CO Isotopes.

Samples were collected weekly at Ragged Point in Barbados in 1996 to 1999, at the top of a 15-m tower, which sits atop a 15-m bluff overlooking the Atlantic. The vast majority of samples were collected during an east-south-east flow in the morning (8:00 to 12:00) with wind speeds in excess of 10 m s^−1^. CO isotope analysis was done with an isotope ratio mass spectrometer. More details on the sample collection protocol and analysis of CO isotopes are provided in Mak et al. (25). Mak et al. (25) data were shown as 3-wk averages, but in our Fig. 1, we show the raw data that have not been published before.

Fig. 1 shows the same inverse model simulations as used by Mak et al. (25), which is an inverse model simulation using TM2 from Bergamaschi et al. (54). It was noted that these model simulations are for a perpetual year reflecting the climatological wind fields. Model simulations were tuned to a set of previously published stable isotope data which were collected from middle- and high-latitude stations in both hemispheres.

We note that perfect agreement between model and observations is not expected as the Mak et al. (25) samples were taken with nominal conditions of 10 m s^−1^ wind from the east-south-east, giving an increased chance of sampling air parcels affected by dust, whereas the model data present δ^13^C-CO values predicted for the region regardless of local meteorological conditions.

### Calculation of Acid Displacement Source Magnitude.

Using the reaction rate constant for HCl + OH, *k*(298 K) = 7.86 × 10^−13^ cm^3^ s^−1^ (55) and a relatively high mole fraction of *x*_HCl_ = 400 ppt HCl (7) and a *x*_OH_ of 0.14 ppt (double the diurnal average of 0.07 ppt, see *SI Appendix*, Fig. S6), we calculate a maximum Cl production rate from acid displacement of 3 × 10^4^ cm^−3^ s^−1^. This is more than 1 order of magnitude below the Cl production rates by MDSA that were needed to explain the Barbados anomaly.

## Supplementary Material

Appendix 01 (PDF)

## Data Availability

Text data have been deposited in University of Copenhagen repository (https://erda.ku.dk/archives/7b38cf06cc971b21faecca41e8924f6b/published-archive.html) (56).

## References

[1] C. J. Young , Chlorine as a primary radical: Evaluation of methods to understand its role in initiation of oxidative cycles. Atmos. Chem. Phys. **14**, 3427–3440 (2014).

[2] IPCC, “Summary for policymakers” in Climate Change 2021: The Physical Science Basis. Contribution of Working Group I to the Sixth Assessment Report of the Intergovernmental Panel on Climate Change (ISBN 978-92-9169-158-6, Cambridge University Press, Cambridge, United Kingdom and New York, NY, USA, 2021), pp. 3–32, doi:10.1017/9781009157896.001.

[3] E. Dlugokencky; NOAA/GML, “Trends in atmospheric methane”. gml.noaa.gov/ccgg/trends_ch4/. Accessed 15 May 2023.

[4] G. Saueressig , Carbon 13 and D kinetic isotope effects in the reactions of CH 4 with O(1 D) and OH: New laboratory measurements and their implications for the isotopic composition of stratospheric methane. J. Geophys. Res. **106**, 23127–23138 (2001).

[5] G. Saueressig, P. Bergamaschi, J. N. Crowley, H. Fischer, G. W. Harris, Carbon kinetic isotope effect in the reaction of CH_4_ with Cl atoms. Geophys. Res. Lett. **22**, 1225–1228 (1995).

[6] W. Allan, D. C. Lowe, A. J. Gomez, H. Struthers, G. W. Brailsford, Interannual variation of ^13^C in tropospheric methane: Implications for a possible atomic chlorine sink in the marine boundary layer. J. Geophys. Res. Atmospheres **110** (2005).

[7] K. L. Feilberg, D. W. Griffith, M. S. Johnson, C. J. Nielsen, The ^13^C and D kinetic isotope effects in the reaction of CH_4_ with Cl. Intern. J. Chem. Kinetics **37**, 110–118 (2005).

[8] T. Röckmann, C. A. M. Brenninkmeijer, P. J. Crutzen, U. Platt, Short-term variations in the ^13^C/^12^C ratio of CO as a measure of Cl activation during tropospheric ozone depletion events in the Arctic. J. Geophys. Res. **104**, 1691–1697 (1999).

[9] U. Platt, W. Allan, D. Lowe, Hemispheric average Cl atom concentration from ^13^C/^12^C ratios in atmospheric methane. Atmos. Chem. Phys. **4**, 2393–2399 (2004).

[10] R. Hossaini , A global model of tropospheric chlorine chemistry: Organic versus inorganic sources and impact on methane oxidation. J. Geophys. Res. Atmos. **121**, 271–297 (2016).

[11] T. Sherwen , Global impacts of tropospheric halogens (Cl, Br, I) on oxidants and composition in GEOS-Chem. Atmos. Chem. Phys. **16**, 12239–12271 (2016).

[12] S. Gromov, C. A. M. Brenninkmeijer, P. Jöckel, A very limited role of tropospheric chlorine as a sink of the greenhouse gas methane. Atmos. Chem. Phys. **18**, 9831–9843 (2018).

[13] Q. Li , Reactive halogens increase the global methane lifetime and radiative forcing in the 21st century. Nat. Commun. **13**, 2768 (2022). 35589794 10.1038/s41467-022-30456-8 PMC9120080

[14] X. Wang , Global tropospheric halogen (Cl, Br, I) chemistry and its impact on oxidants. Atmos. Chem. Phys. **21**, 13973–13996 (2021).

[15] S. Schwietzke , Upward revision of global fossil fuel methane emissions based on isotope database. Nature **538**, 88–91 (2016). 27708291 10.1038/nature19797

[16] E. G. Nisbet , Rising atmospheric methane: 2007–2014 growth and isotopic shift. Global Biogeochem. Cycles **30**, 1356–1370 (2016).

[17] H. Schaefer , A 21st-century shift from fossil-fuel to biogenic methane emissions indicated by ^13^CH_4_. Science **352**, 80–84 (2016). 26966190 10.1126/science.aad2705

[18] S. A. Strode , Strong sensitivity of the isotopic composition of methane to the plausible range of tropospheric chlorine. Atmos. Chem. Phys. **20**, 8405–8419 (2020).

[19] X. Lan , Improved constraints on global methane emissions and sinks using δ^13^C-CH_4_. Global Biogeochem. Cycles **35**, e2021GB007000 (2021). 10.1029/2021GB007000 PMC8244052 34219915

[20] J. Thanwerdas , How do Cl concentrations matter for the simulation of CH4 and δ^13^C(CH_4_) and estimation of the CH_4_ budget through atmospheric inversions? Atmos. Chem. Phys. **22**, 15489–15508 (2022).

[21] T. Nukurangi, National Institute of Water and Atmospheric Research (NIWA), June 2022. https://niwa.co.nz/atmosphere/our-data/trace-gas-plots/methane (2022). Accessed 5 March 2023.

[22] K. A. Read , Intra-annual cycles of NMVOC in the tropical marine boundary layer and their use for interpreting seasonal variability in CO. J. Geophys. Res. Atmos. **114**, D21303 (2009).

[23] M. J. Lawler , HOCl and Cl_2_ observations in marine air. Atmos. Chem. Phys. **11**, 7617–7628 (2011).

[24] P. Bonasoni , Aerosol-ozone correlations during dust transport episodes. Atmosp. Chem. Phys. **4**, 1201–1215 (2004).

[25] J. E. Mak, G. Kra, T. Sandomenico, P. Bergamaschi, The seasonally varying isotopic composition of the sources of carbon monoxide at Barbados, West Indies. J. Geophys. Res. Atmos. **108**, 4635 (2003).

[26] S. Tilmes , Representation of the community earth system model (CESM1) CAM4-chem within the chemistry-climate model initiative (CCMI). Geosci. Model Dev. **9**, 1853–1890 (2016).

[27] J.-F. Lamarque , CAM-chem: Description and evaluation of interactive atmospheric chemistry in the community earth system model. Geosci. Model Dev. **5**, 369–411 (2012).

[28] M. O. Andreae , Internal mixture of sea salt, silicates, and excess sulfate in marine aerosols. Science **232**, 1620–1623 (1986). 17812139 10.1126/science.232.4758.1620

[29] S. E. Harnung, M. S. Johnson, Chemistry and The Environment (Cambridge University Press, 2012).

[30] X. Zhu, J. M. Prospero, F. J. Millero, D. L. Savoie, G. W. Brass, The solubility of ferric ion in marine mineral aerosol solutions at ambient relative humidities. Mar. Chem. **38**, 91–107 (1992).

[31] J. Wittmer, C. Zetzsch, Photochemical activation of chlorine by iron-oxide aerosol. J. Atmos. Chem. **74**, 187–204 (1992).

[32] T. K. Koenig , Ozone depletion due to dust release of iodine in the free troposphere. Sci. Adv. **7**, eabj6544 (2021). 34936464 10.1126/sciadv.abj6544 PMC8694599

[33] X. Zhu , Photoreduction of iron(III) in marine mineral aerosol solutions. J. Geophys. Res. **98**, 9039–9046 (1993).

[34] X. R. Zhu, J. M. Prospero, F. J. Millero, Diel variability of soluble Fe(II) and soluble total Fe in North African dust in the trade winds at Barbados. J. Geophys. Res. **102**, 21297–21305 (1997).

[35] Y. Chen, R. L. Siefert, Seasonal and spatial distributions and dry deposition fluxes of atmospheric total and labile iron over the tropical and subtropical North Atlantic Ocean. J. Geophys. Res. **109**, D09305 (2004).

[36] J. Wittmer, S. Bleicher, C. Zetzsch, Iron(III)-Induced activation of chloride and bromide from modeled salt pans. J. Phys. Chem. A **119**, 4373–4385 (2015). 25243918 10.1021/jp508006s

[37] X. Wang , The role of chlorine in global tropospheric chemistry. Atmos. Chem. Phys. **19**, 3981–4003 (2019).

[38] D. L. Savoie , Marine biogenic and anthropogenic contributions to non-sea-salt sulfate in the marine boundary layer over the North Atlantic Ocean. J. Geophys. Res. Atmos. **107**, AAC-3 (2002).

[39] M. Bräunlich, Study of Atmospheric Carbon Monoxide and Methane using Isotopic Analysis (Ruprecht-Karls-Univ, Heidelberg, Germany, 2000).

[40] C. Tsamalis, A. Chédin, J. Pelon, V. Capelle, The seasonal vertical distribution of the Saharan Air Layer and its modulation by the wind. Atmos. Chem. Phys. **13**, 11235–11257 (2013).

[41] M. van der Does, L. F. Korte, C. I. Munday, G.-J.A. Brummer, J.-B.W. Stuut, Particle size traces modern Saharan dust transport and deposition across the equatorial North Atlantic. Atmos. Chem. Phys. **16**, 13697–13710 (2016).

[42] C. A. Cuevas , The influence of iodine on the Antarctic stratospheric ozone hole. Proc. Natl. Acad. Sci. U.S.A. **119**, e2110864119 (2022). 35131938 10.1073/pnas.2110864119 PMC8851550

[43] N. M. Mahowald , Observed 20th century desert dust variability: Impact on climate and biogeochemistry. Atmos. Chem. Phys. **10**, 10875–10893 (2010).

[44] P. Zuidema , Is summer African dust arriving earlier to Barbados? The updated long-term in situ dust mass concentration time series from Ragged Point, Barbados, and Miami, Florida. Bull. Am. Meteorol. Soc. **100**, 1981–1986 (2019).

[45] T. Yuan , Anthropogenic decline of African dust: Insights from the Holocene records and beyond. Geophys. Res. Lett. **47**, e2020GL089711 (2020). 10.1029/2020GL089711 PMC7685148 33281243

[46] A. M. Johansen, R. L. Siefert, M. R. Hoffmann, Chemical composition of aerosols collected over the tropical North Atlantic Ocean. J. Geophys. Res. **105**, 15277–15312 (2000).

[47] Y. Chen, R. L. Siefert, Determination of various types of labile atmospheric iron over remote oceans. J. Geophys. Res. **108**, 4774 (2003).

[48] J. M. Trapp, F. J. Millero, J. M. Prospero, Trends in the solubility of iron in dust-dominated aerosols in the equatorial Atlantic trade winds: Importance of iron speciation and sources. Geochem. Geophys. Geosyst. **11**, Q03014 (2010).

[49] J. Wittmer, S. Bleicher, J. Ofner, C. Zetzsch, Iron(III)-induced activation of chloride from artificial sea-salt aerosol. Environ. Chem. **12**, 461 (2015).

[50] H. O. Pye , The acidity of atmospheric particles and clouds. Atmos. Chem. Phys. **20**, 4809–4888 (2020). 33424953 10.5194/acp-20-4809-2020 PMC7791434

[51] E. R. Sholkovitz, P. N. Sedwick, T. M. Church, A. R. Baker, C. F. Powell, Fractional solubility of aerosol iron: Synthesis of a global-scale data set. Geochim. Cosmochim. Acta **89**, 173–189 (2012).

[52] N. M. Mahowald , Aerosol trace metal deposition dissolution and impacts on marine microorganisms and biogeochemistry. Nat. Commun. **81**, 1–15 (2018). 10.1038/s41467-018-04970-7 PMC6033952 29977041

[53] G. G. Pfister , Contribution of isoprene to chemical budgets: A model tracer study with the NCAR CTM MOZART-4. J. Geophys. Res. **113**, D05308 (2008).

[54] P. Bergamaschi, R. Hein, C. A. M. Brenninkmeijer, P. J. Crutzen, Inverse modeling of the global CO cycle: 2. Inversion of ^13^C/^12^C and ^18^O/^16^O isotope ratios. J. Geophys. Res. **105**, 1929–1945 (2000).

[55] R. Atkinson , Evaluated kinetic and photochemical data for atmospheric chemistry: Volume III–gas phase reactions of inorganic halogens. Atmos. Chem. Phys. **7**, 981–1191 (2007).

[56] M. M. J. W. van Herpen , Supplementary data for Photocatalytic Chlorine Atom Production on Mineral Dust-Sea Spray Aerosols over North Atlantic. Electronic Research Data Archive. https://erda.ku.dk/archives/5ae49a1d98c54406adb0f4e4b31b8106/published-archive.html. Deposited 6 July 2023. 10.1073/pnas.2303974120 PMC10400977 37487065

