# Supplementary material for: Photocatalytic chlorine atom production on mineral dust–sea spray aerosols over the North Atlantic
Source: Proc Natl Acad Sci U S A. 2023 Jul 24;120(31):e2303974120. doi: 10.1073/pnas.2303974120 (PMC10400977; doi:10.1073/pnas.2303974120)
Supplement: Supplementary file 1 — Appendix 01 (PDF) [file pnas.2303974120.sapp.pdf]

PNAS

Supporting Information for

Photocatalytic Chlorine Atom Production on Mineral Dust-Sea Spray Aerosols over North Atlantic

Maarten M.J.W. van Herpen[1], Qinyi Li[2,3], Alfonso Saiz-Lopez[2], Jesper B. Liisberg[4], Thomas Röckmann[5], Carlos A. Cuevas[2], Rafael P. Fernandez[6], John E. Mak[7], Natalie M. Mahowald[8], Peter Hess[8], Daphne Meidan[8], Jan-Berend W. Stuut[9,10], Matthew S. Johnson[4*]

*Corresponding author: Matthew S. Johnson.

Email: msj@chem.ku.dk

Supporting Information Text

Limitations of the model parameters

Our model parameterization used reaction rate constants for iron photochemistry that were determined for mineral dust at Barbados by experiment *(1)*, and the model used photoactive iron concentrations from independent observations, including from Barbados *(2)*. The results of the field study could presumably also be explained with different combinations of parameters e.g. by using a lower yield combined with a higher rate.

Because the input to the model are observations from across the North Atlantic at surface level, we believe it should give a good description of the CO isotope anomaly at Barbados. More observations are needed to determine whether the model parameters also apply to other regions, and also what parameters might be needed for high altitude.

The reaction rate for photo-oxidation of Fe(II) was estimated based on the observation that it is not the limiting step for photochemical cycling. The uncertainty in this reaction rate could introduce an error of +50 to -10 % based on changing the photoreduction rate to the limiting values i.e. either equal to the oxidation rate or set to zero. It is also possible that not every Fe(II) – Fe(III) cycle leads to the production of Cl (we assumed a yield of 100 %).

There are alternative reactions that could produce the photo-chemical reduction of Fe(III), including organics (leading to H_2O_2) *(1)*, but we assumed that the dominant photoreduction reaction in MDSA produces Cl and/or OH *(3)* both of which lead to Cl_2 production. Some side reactions in a complex system like seawater and dust will almost certainly reduce the rate and limit the amount Cl(0) production; Wittmer has noted that the presence of SO_4^{-2} inhibits the formation of Cl_2 *(4,5)*. This could mean that our model overestimates Cl production. Further research on the mechanism is indicated. However, the resulting cycle rate was on the low end of the range of values reported by Wittmer *(3)*, 11 hr^{-1} in our model versus 6-78 hr^{-1} by Wittmer, which could mean that our model is underestimating Cl production.

Reactive bromine is very effective at ozone destruction, and therefore relevant for our conclusions about methane lifetime. However, Wittmer *(6)* measured the Cl and Br production from artificial sea-salt aerosols mixed with $FeCl_3$ in a smog chamber, and found high Cl production rates, while Br production rates remained low, except under very high O_3 background concentrations of 700 ppb. This is consistent with the finding in our model simulation that the significant reduction in O_3 in the marine boundary layer resulted in a reduction in bromine and iodine emission and their atmospheric abundance. The sources of reactive bromine in the CESM model mainly include the

bromine-induced reactions (e.g., HOBr and $BrONO_2$ uptake on sea-salt aerosol) and the oceanic biogenic releases of organic bromines (e.g., $CHBr_3$). For the iodine, the main sources include the O_3 deposition onto the oceanic surface (activating the releases of HOI and I_2 from ocean surface) and the oceanic biogenic releases of organic iodines. The model does not include detailed aerosol chemistry, such as the reaction of HOCl with bromine-containing aerosol.

Our model parameterization does not take into account differences in photoactive iron concentrations for other mineral dust sources, and does not take into account potential changes in photoactive iron during downwind transport or anthropogenic dust emissions. This will lead to further uncertainty Cl production in other world regions. For the North Atlantic it might affect the model results close to the African coast (since we used observations further from Africa for our assumptions), but it will have only a small impact on the model results at Barbados.

Our model simulation only included the production of MDSA up to an altitude of 900 hPa. Above this altitude, humidity and concentration of sea spray and dust aerosols are very different. We have not found evidence of photo-active iron mixed with sea-salt in mineral dust in this region or at these conditions. Further research should investigate whether MDSA-Cl is also generated at these high altitudes.

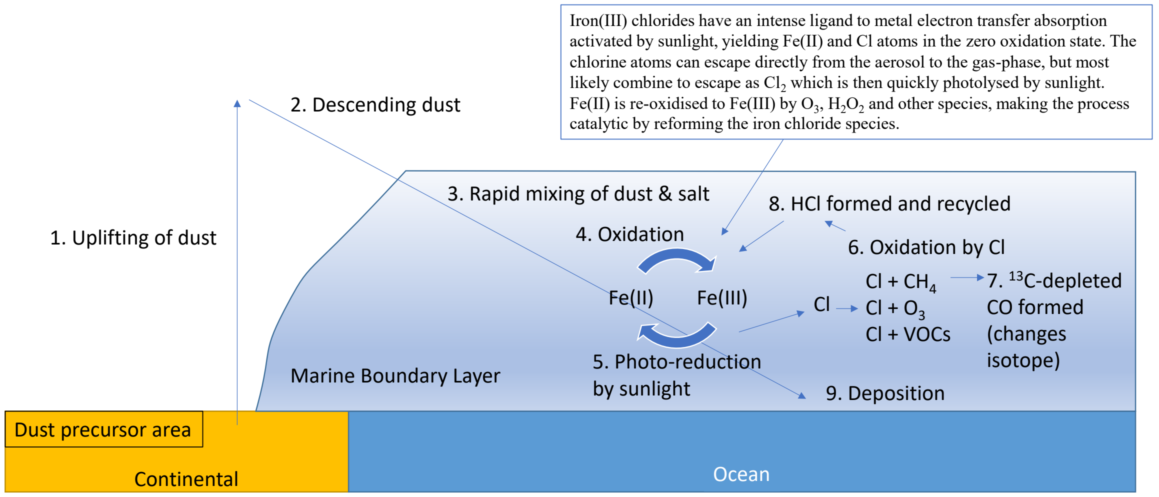

Fig. S1. Schematic overview of the MDSA mechanism.

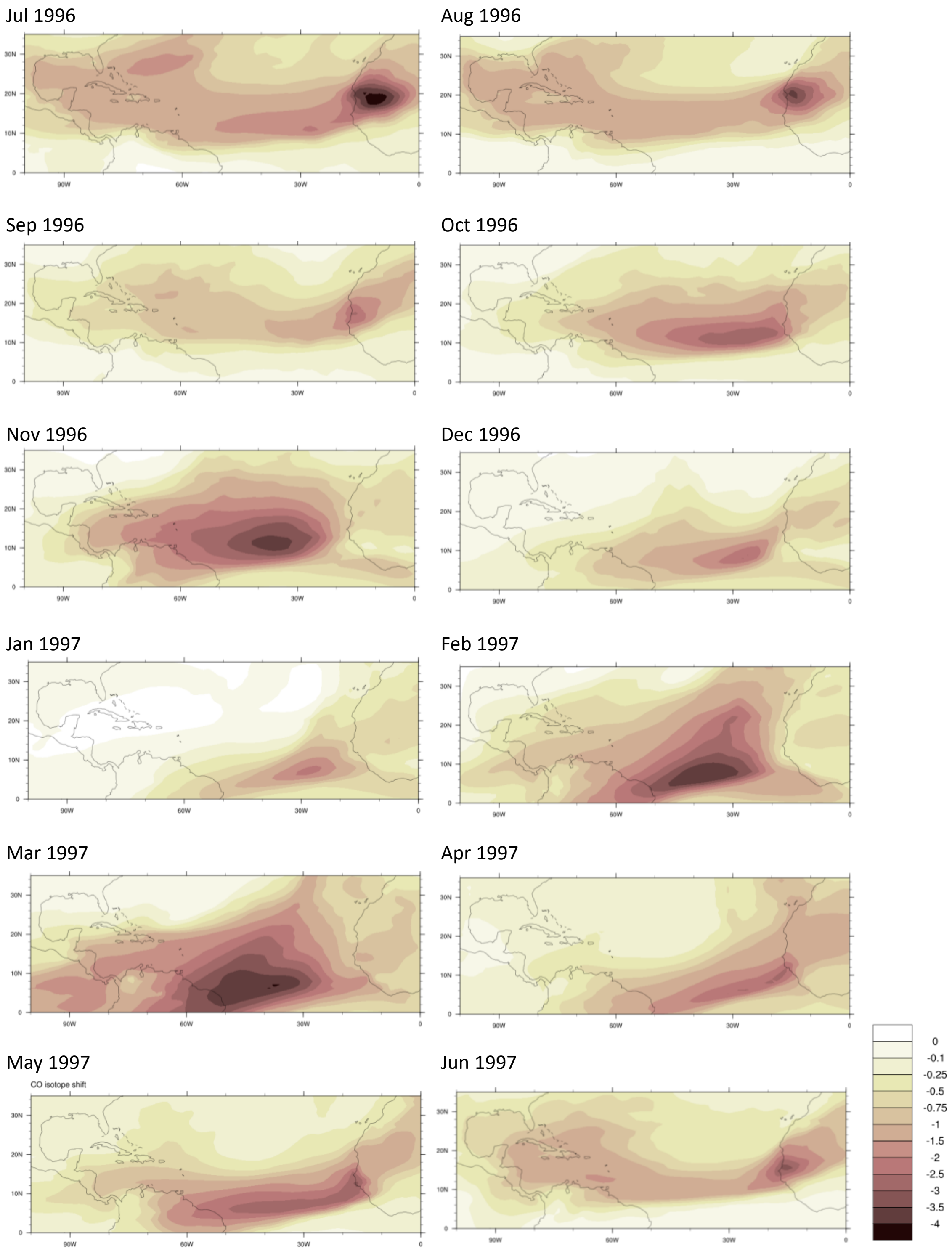

Fig. S2. Monthly average ^{13}C in CO change due to MDSA, for Jul 1996 - Jun 1997.

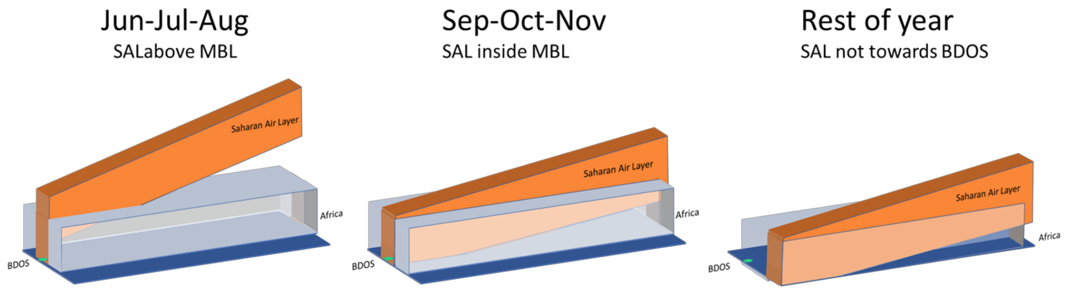

Fig. S3. Schematic showing the different transport heights and direction through the year, for the Saharan Air Layer (SAL) through the marine boundary layer (MBL) towards Barbados (BDOS).

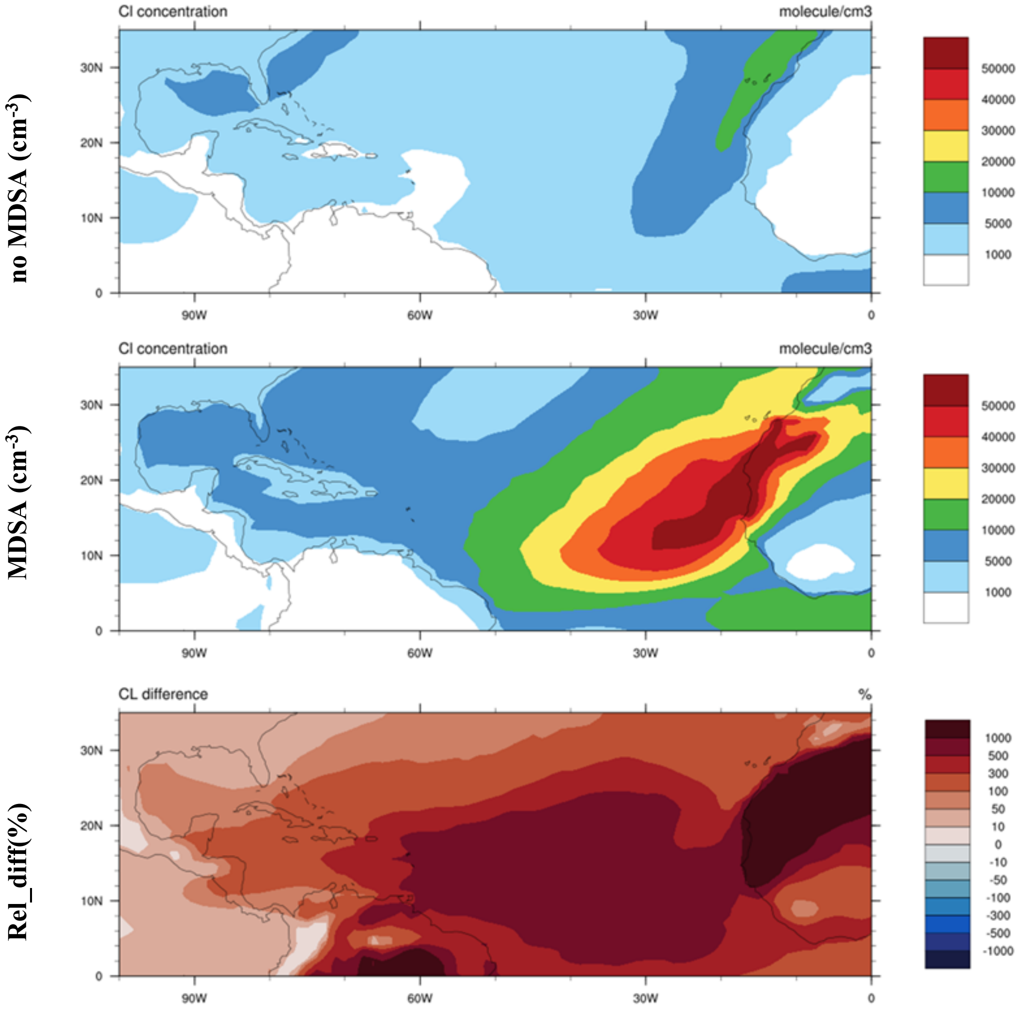

Fig. S4. Spatial pattern of Cl number concentrations (cm^{-3}) and their changes (%) due to MDSA-induced chlorine emission within the marine boundary layer in the North Atlantic. The absolute change (ppqv) is shown in Fig. 1a.

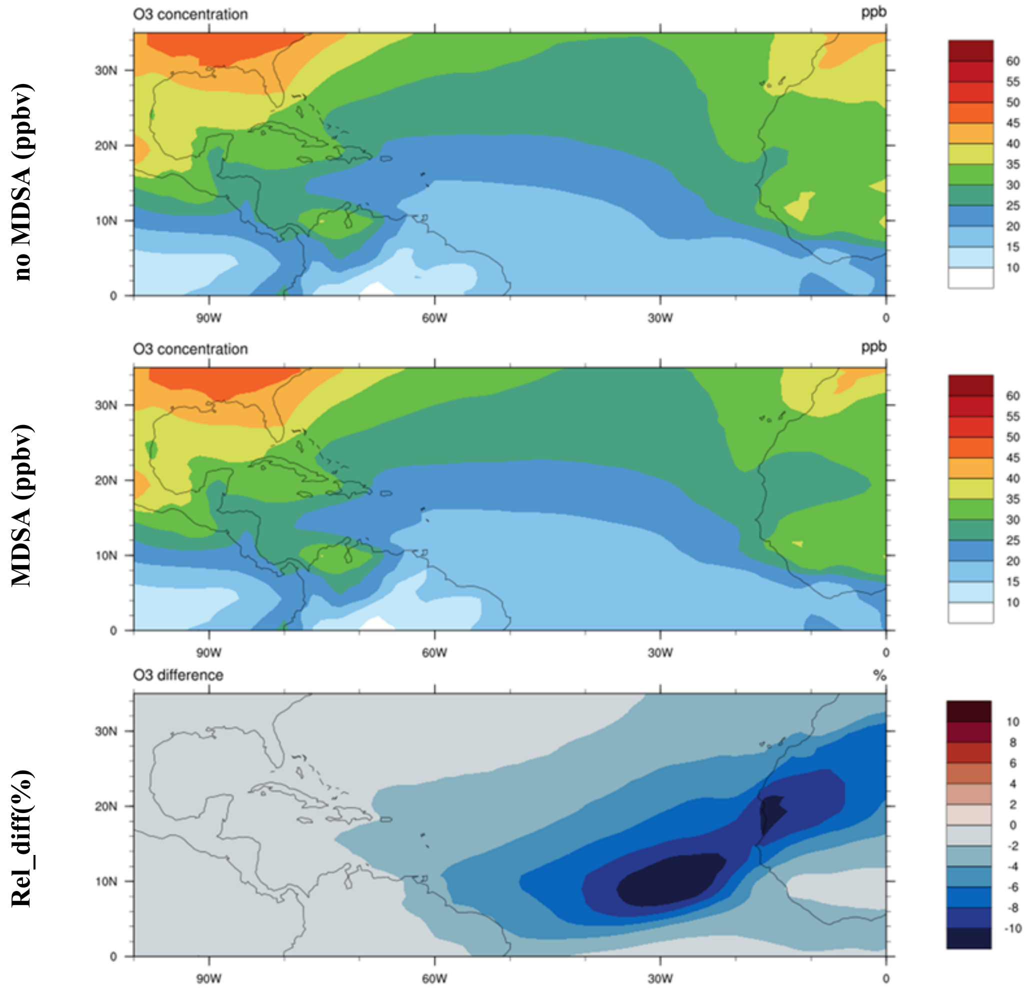

Fig. S5. Spatial pattern of O_3 mixing ratios (ppbv) and their changes (%) due to MDSA-induced chlorine emission within the marine boundary layer in the North Atlantic. The absolute change (ppbv) is shown in Fig. 1b.

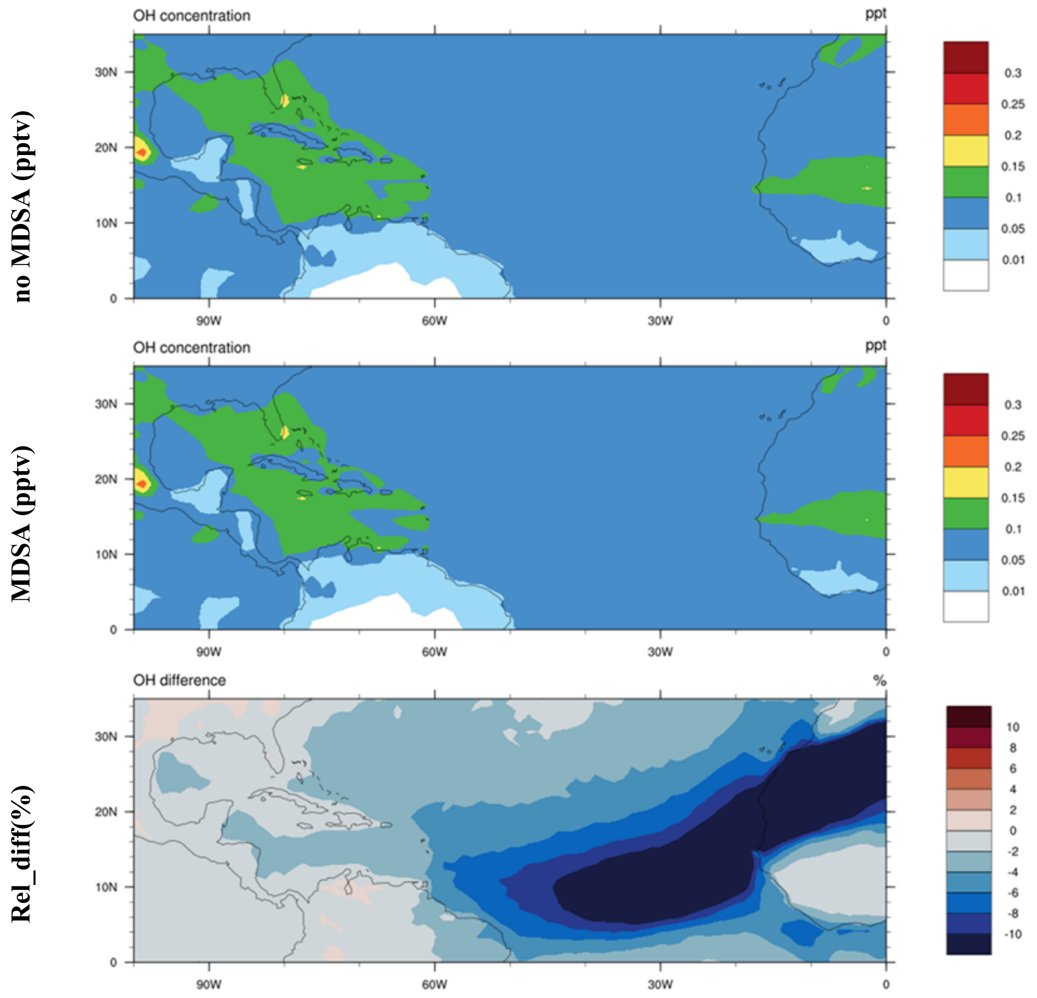

Fig. S6. Spatial pattern of OH mixing ratios (pptv) and their changes (%) due to MDSA-induced chlorine emission within the marine boundary layer in the North Atlantic. The absolute change (pptv) is shown in Fig. 1c.

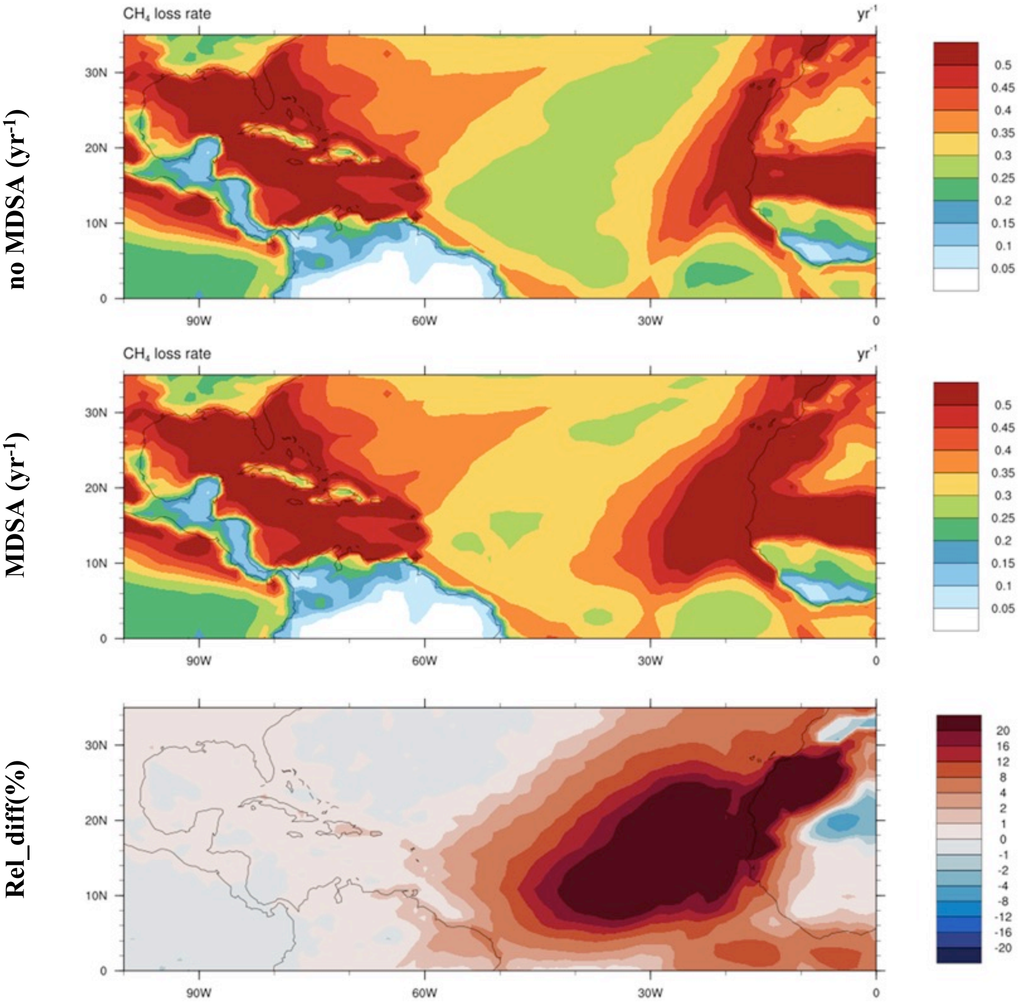

Fig. S7. Spatial pattern of CH_4 loss rates (yr^{-1}) and their changes (%) due to MDSA-induced chlorine emission within the marine boundary layer in the North Atlantic. The absolute change (yr^{-1}) is shown in Fig. 1d.

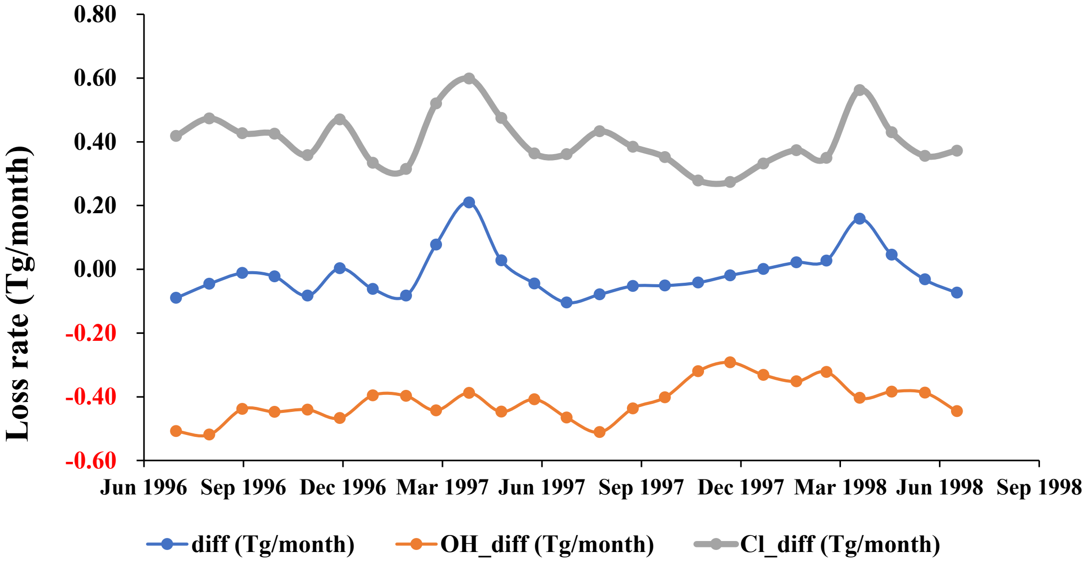

Fig. S8. Simulated change due to MDSA in global integrated CH_4 loss rate by tropospheric OH, by tropospheric chlorine, and in total (blue line). For a full year, the total CH_4 loss rate was reduced (by 0.01 Tg/month) by MDSA, suggesting that the global burden of CH_4 is slightly increased.

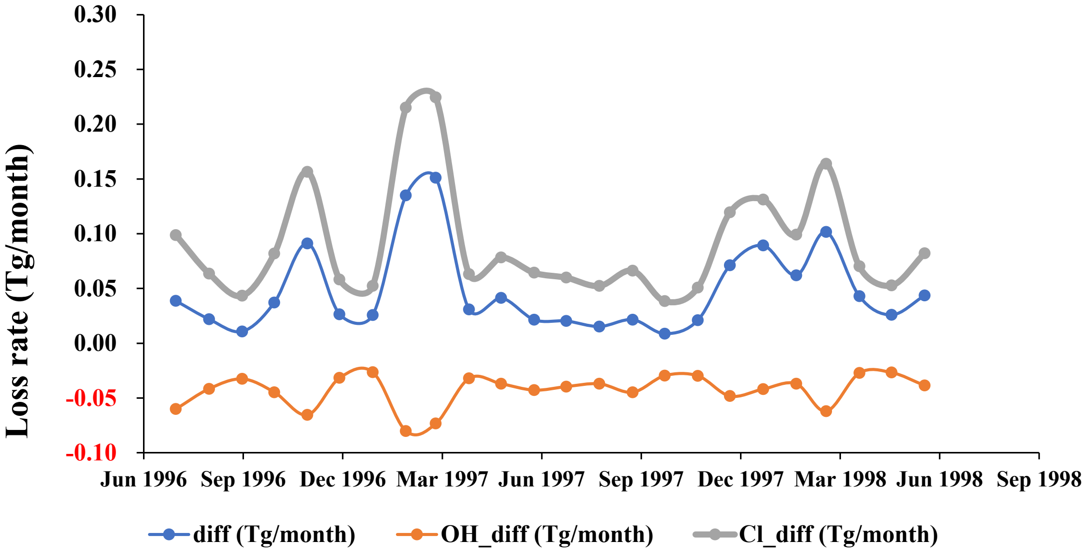

Fig. S9. The same as Fig. S5 but for the marine boundary layer (excluding the continental regions) in the North Atlantic. In this case the total CH_4 loss rate (blue line) is increased. The annual average result is shown in Fig. 2 in the main text.

Table S1. Key parameters of the ISA process for the North Atlantic boundary layer (BL) and the global atmosphere.

Region	Case	Fe burden in boundary layer / Gg		MDSA induced Cl_2 production in BL / ($Tg\ yr^{-1}$)		O_3 burden [a] / Tg	Cl contribution to CH_4 loss [a]		OH contribution to CH_4 loss [a]	
		Marine	Marine & Continental	Marine	Marine & Continental		$Tg\ yr^{-1}$	%	$Tg\ yr^{-1}$	%
North Atlantic [b]	No MDSA	0.22	0.49	-		1.6	0.3	2.7	11.4	97.3
	MDSA			2.89	3.79	1.5	1.4	11.4	10.9	88.5
Global	No MDSA	0.60	2.34	-		305.6	21.9	4.0	506.8	93.8
	MDSA			8.09	13.12	303.5	26.7	4.9	501.9	92.9

(a) Boundary layer values are used for the North Atlantic defined as surface to 900 hPa pressure. For the global atmosphere the area from the surface to the tropopause is considered defined as the layer with >150 ppbv O_3.

(b) The North Atlantic is defined as the ocean region with latitude between 0 and 35°N and longitude between 0 and 100°W, as in Fig. 2 but excluding land.

Table S2.

Simulation design of CAM-Chem model.

Case	Dust induced Cl_2 production	Tropospheric chlorine chemistry
noMDSA	No	Yes
MDSA	Yes	Yes

Table S3.
Simulated O_3 loss (normalized by O_3 concentrations) from each main channels in the Northern Atlantic in MBL and their changes induced by MDSA, s^{-1}.

Family	noMDSA / s^{-1}	MDSA / s^{-1}	Absolute Change / s^{-1}	Relative Change /%
O_x	1.22×10^{-6}	1.23×10^{-6}	8.79×10^{-9}	0.72
HO_x	5.12×10^{-7}	5.09×10^{-7}	-2.81×10^{-9}	-0.55
NO_x	1.27×10^{-8}	1.23×10^{-8}	-3.71×10^{-10}	-2.92
ClO_x	1.21×10^{-8}	5.97×10^{-8}	4.76×10^{-8}	392.68
BrO_x	1.33×10^{-7}	1.17×10^{-7}	-1.66×10^{-8}	-12.43
IO_x	4.79×10^{-7}	4.78×10^{-7}	-5.68×10^{-10}	-0.12
ClO_x-BrO_x	9.72×10^{-9}	1.86×10^{-8}	8.90×10^{-9}	91.54
Sum	2.38×10^{-6}	2.42×10^{-6}	4.49×10^{-8}	1.89

Note: We follow *(7)* to categorize the O_3 loss channels.

Supplementary References

1. Zhu, X. *et al.*, Photoreduction of iron(III) in marine mineral aerosol solutions. *J. Geophys. Res.* **98**(D5), 9039– 9046 (1993).
2. Zhu, X. R., Prospero, J. M., and Millero, F. J., Diel variability of soluble Fe(II) and soluble total Fe in North African dust in the trade winds at Barbados. *J. Geophys. Res.*, **102**(D17), 21297–21305 (1997).
3. Wittmer, J. and Zetzsch, C., Photochemical activation of chlorine by iron-oxide aerosol. *Journal of Atmospheric Chemistry* **74**(2), 187-204 (2017).
4. Wittmer, J., Bleicher, S.,, and Zetzsch, C., Iron(III)-Induced Activation of Chloride and Bromide from Modeled Salt Pans. *The Journal of Physical Chemistry A* **119** (19), 4373-4385, (2015).
5. Trapp, J. M., Millero, F. J., and Prospero, J. M., Trends in the solubility of iron in dust-dominated aerosols in the equatorial Atlantic trade winds: Importance of iron speciation and sources. *Geochem. Geophys. Geosyst*. **11**, (2010).
6. Wittmer, J., Bleicher, S., Ofner, J., Zetzsch, C. Iron(III)-induced activation of chloride from artificial sea-salt aerosol. *Environmental Chemistry* **12**(4), 461 (2015).
7. Cuevas, C.A. et al., The influence of iodine on the Antarctic stratospheric ozone hole. *Proceedings of the National Academy of Sciences* **119**(7), e2110864119 (2022).
